# Factors influencing utilization of communicable disease prevention and treatment education among the floating population: a cross-sectional study in China

**DOI:** 10.1186/s12889-023-15126-8

**Published:** 2023-01-31

**Authors:** Xiaodan Lin, Xiuhua Mao, FuZhi Ai, Weiguang Yao

**Affiliations:** 1 School of Health Management, Southern Medical University, Guangzhou, People’s Republic of China; 2 Department of Orthopaedics, Sun Yat-sen Memorial Hospital, Sun Yat-sen University, Guangzhou, People’s Republic of China

**Keywords:** Anderson health service utilization model, Communicable disease health literacy, Health education, Associated factors, Migrant

## Abstract

**Background:**

In China, communicable diseases (CD) have a negative impact on public health and economic stability. The influx of migrants, who make up a substantial portion of China’s population and continue to rapidly expand, has seriously hampered CD prevention and control, needing special care. This study aimed to identify key factors influencing the utilization of CD prevention and treatment education (CDPTE) among the floating population. We are confident that the findings will highlight obstacles facing CDPTE among the migrants, and guide future development prevention, treatment of CD, and health education services.

**Methods:**

A sample of migrants aged 15 years and above in 32 provincial units nationwide in 2018 was recruited by stratified multi-stage proportional to population size sampling (PPS). A structured questionnaire survey was conducted via face-to-face interviews. Subsequently, the Anderson health service utilization model was used as the theoretical framework and SPSS 26.0 statistical software was applied to analyze the data. The statistical description of the current situation of CDPTE acceptance and the chi-square test were used to compare the differences in CDPTE acceptance by different characteristics. Multivariate logistic regression was used to analyze key factors affecting the use of CDPTE among migrants.

**Results:**

A total of 40.1% of the recruited participants reported receiving education on CD prevention and treatment, primarily delivered through traditional transmission media. Multilevel logistic regression results revealed that male migrants, aged 30–49 years, unmarried, with higher educational attainment, an average monthly household income of CNY 7,500-9,999 (or US$1,176-1,568), working more than 40 h per week, flowing into the Central and Western regions, migrated in the province, self-rated health, contracted family doctors and those with health records were more likely to receive CDPTE (*p* < 0.05).

**Conclusion:**

Our findings revealed unsatisfactory acceptance of education on CD prevention and treatment among migrants, implying that health education should be strengthened further. Publicity of relevant policies and works should be strengthened and specific interventions should be developed for key regions as well as vulnerable groups to enhance CDPTE. More financial support should also be provided to improve the quality of health education.

## Background

Communicable diseases (CDs) are a class of diseases caused by various pathogens that can be transmitted from human to human, animal to animal, or human to animal, and are transmissible and epidemic [1]. CDs are a leading cause of death and disabilities in humans, posing a serious burden on public health and economic stability [2]. According to Ten threats to global health 2019, diseases and behaviors such as the global influenza pandemic, Ebola and other high-threat pathogens, vaccines, HIV, and other diseases closely related to CDs account for half of the threats [3]. According to World Health Organization (WHO), an estimated 228 million cases of malaria and 602,000 deaths across the globe were reported in 2021 [4]. In addition, 650,000 global deaths from AIDS-related illnesses were still reported, and 6% less international funding was available for the AIDS response than in 2010 [5]. In recent years, China has experienced sudden outbreaks of new CDs including atypical pneumonia, avian-human influenza, H1N1 influenza, and coronavirus disease 2019 (COVID-19), which have severely affected the lives of residents, disrupted societal order, and even threatened the health and lives of the population [6]. According to the National Report of Statutory Infectious Diseases in 2021 [7], 6,233,537 cases of statutory CDs and 22,198 deaths were reported nationwide (excluding Hong Kong, Macao Special Administrative Region, and Taiwan), with an incidence rate of 442.16/100,000 and a mortality rate of 1.57/100,000. This indicates that global nations, including China, have weak prevention and control strategies for CDs. With the increasing development of transportation and logistics, the mobility and congregation of people have increased. As a result, the probability of global transmission of various major CDs has significantly increased [8, 9], and CDs remain a constant threat to human life and even affect social development. CDPTE has significant relevance and practical value; strengthening the capacity of the public to prevent and control epidemics, as well as improving the health literacy of CD, have become essential priorities for government and issues of social concern.

At the founding conference of the United Nations in 1945, delegates called for the establishment of the WHO to coordinate global health policies and actions, particularly in response to global or regional CDs [10]. The Chinese health authorities positively responded to this request, and developed strategies focused on the at-risk populations, including migrants [11]. Both the “New Medical Reform” in 2009 and the “Healthy China 2030 Strategy” in 2016 proposed strengthening the construction of the public health system and improving monitoring and prevention and control of major CDs to improve CD surveillance and early warning mechanisms [12, 13], and effectively reduce the spread of CDs such as cholera, dysentery, febrile, malaria, black sickness, schistosomiasis [14]. China is reportedly the country with the largest number of internal migrants globally [15]. This phenomenon is primarily attributed to an increase in urbanization, which subsequently promotes the expansion of migration, thereby significantly promoting urban socio-economic development and social stability. According to the Communiqué of the Seventh National Census of China, the number of migrants in China reached 376 million, accounting for 26.64% of the total population [16]. However, migrants often encounter several obstacles in accessing basic public health and health education services because of irregular immigration status, language barriers, restrictive health-related policies, and economic and social marginalization [17, 18]. Several studies have demonstrated that migrants in China have poor access to public health services compared to permanent residents. This phenomenon has been implicated in the occurrence of many health problems, including CDs, reproductive diseases, occupational diseases, environmental diseases, and psycho-psychiatric disorders [19], which in turn influence employment income, the decision to return home, and urban integration. Reports indicate that the literacy of CD prevention in China has been hovering at a low level, with 26.04% of migrants exhibiting symptoms of CDs in at least one diarrhea, fever, rashes, and jaundice [20]. Migrants are a population with a high prevalence of CDs, and a high-risk group for cross-regional transmission of CDs. This is considered a key constraint to the prevention and control of CDs [21]. Consequently, both their health status and awareness have a considerable effect on social stability and public health in China.

Previous studies have mostly focused on the health needs and use of health services among migrants across different countries or regions, including Germany [22], the United States [23], and Brazil [24]. Additional research has investigated these parameters in specific populations, including pregnant women [25], migrant workers [26], adults with psychiatric disorders [27], and people living with HIV [28]. In China, diverse studies on the health problems of migrants have been reported. Most scholars have primarily explored the health service’ seeking behavior [29], the establishment of health records [30], current status or factors influencing health education [31], and subsequently revealed inequalities in health demands and health services utilization among migrants [17]. So far, only a handful of studies have however assessed the effect of education on CD prevention and treatment among migrants.

Health education is a key element in health promotion. It affects the accessibility of health services and individual self-care capabilities through six main forms, including health knowledge lectures, promotional materials, health education bulletin boards/electronic displays, public health consultation activities, community SMS/WeChat/website, and individualized face-to-face consultation [20]. CDPTE plays an important role in health education. Education on CD prevention and control can guide migrants to improve their self-protection [32], and their capacity to understand and apply knowledge regarding the occurrence, prevention, and treatment of CDs. Consequently, this promotes their health and reduces the occurrence of CDs, as well as regulates the prevention and treatment of CDs among migrants. As highlighted above, focusing on the health level of migrants, coupled with improving prevention and treatment of CDs among them is imperative to effectively meet their needs and an urgent requirement for efficient implementation of the Health China Strategy. This strategy is not only an important objective for regular prevention and control of COVID-19 but also a powerful approach for dealing with unknown challenges facing the prevention and control of CDs in the future. The results are not only expected to better strengthen the prevention and treatment methods of CD and related health education services, but also promote gradual equalization of basic public health services.

## Methods

### Analytic framework

**Fig. 1:**
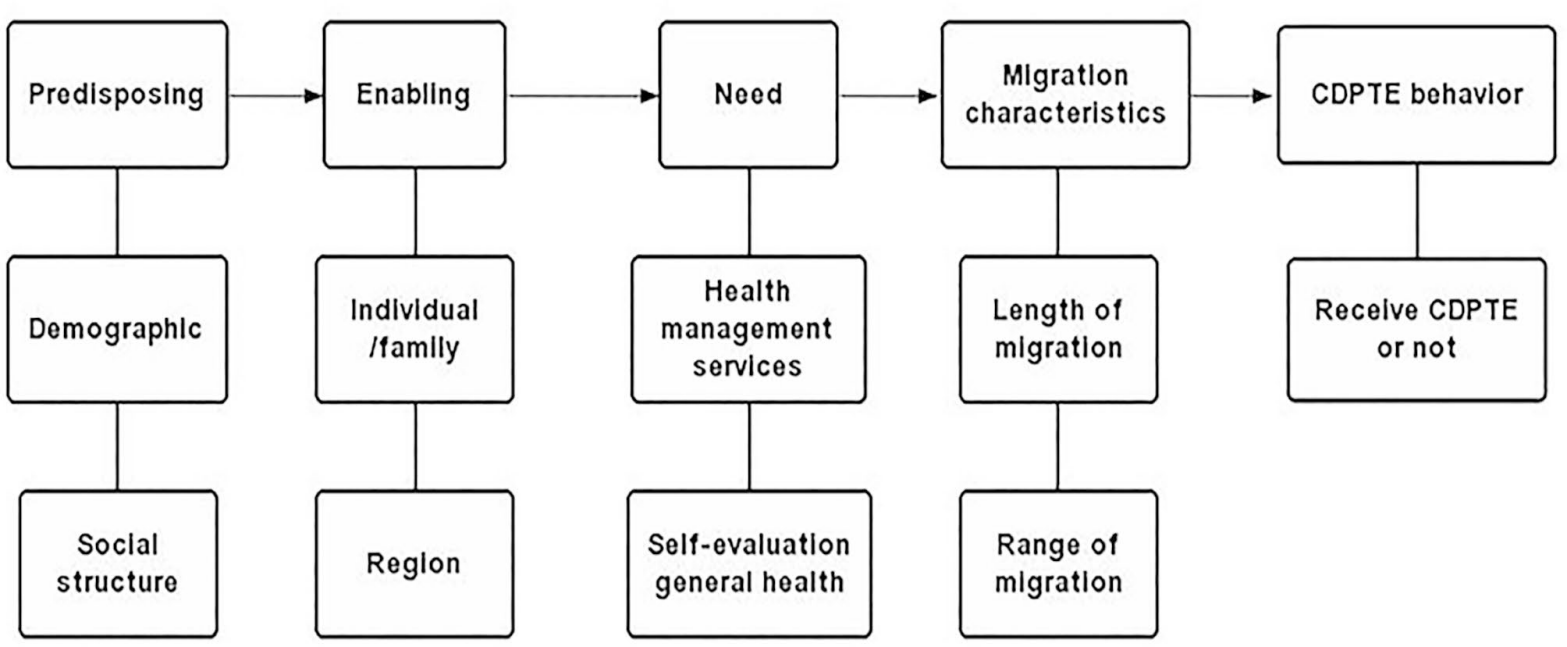
The simplified Anderson health service utilization model: the utilization of CDPTE (receive CDPTE or not) is determined by four dynamics: predisposing factors (demographic and social structure), enabling factors (individual, family and region resources), the need of care (health record, family doctor and self-evaluation general health) and migration characteristics (length of migration and range of migration)

The Anderson health service utilization model, created by Ronald M. Andersen in 1968 [33], is a well-validated theoretical framework for identifying factors affecting health services utilization. The framework takes considers both societal and individual factors from the perspective of systematic analysis [34]. According to the model, health education utilization is determined by three dynamics, including predisposing, enabling, and need variances (PEN). Predisposing factors are personality characteristics, including demographic and socio-structural characteristics, that migrants tend to have to receive health education services. Enabling resources are factors that either promote or impede the availability of personal health services, including support from individual/family resources, and regional resources. Need factors are both self-perceived and actual needs for health education services. Based on previous studies [35, 36] and the evolution as well as adaptation of the Anderson health service utilization model, migration characteristics have the potential to directly or indirectly influence the utilization of health services. For example, migration characteristics, including years of residence and reasons for migration, represent potential pathways through which migration can affect the utilization of basic health services [36]. Therefore, we additionally included migration characteristics as key dynamics parameters and integrated them into the analytical model. A feedback loop was used to illustrate the relationship between CDPTE behaviors (receive CDPTE, and non-receive CDPTE) and other aspects (Fig. 1). We hypothesize that the PEN variables and the migration characteristics variable will be significant predictors of CDPTE use.

### Data sources and research sample

Data were obtained from the 2018 China Migrants Dynamic Survey (CMDS), i.e., a cross-sectional and nationally representative survey conducted annually by the Migrant Population Service Center of the People’s Republic of China (PRC) [20]. Summarily, CMDS collects annual national data of migrants from each province, as the sampling frame, using a stratified, multi-stage, scale-oriented probability proportionate to size (PPS) method as the sampling method. The survey area covered 31 provinces, municipalities, autonomous regions, and migrant gathering areas in Xinjiang Production and Construction Corps across China. A total of 152,000 subjects were recruited. The participants who were aged above 15 years, had moved to the recent living area more than 1 month, but without a local hukou (permanent residence permit). The CMDS contained the basic demographic information of respondents and their household members, as well as comprehensive information on employment, health, and human services status. Further details on the sample are provided elsewhere [37]. The survey was conducted through a face-to-face interview, with all participants voluntarily signing a written informed consent before inclusion in the study. Considering the delay in receiving CDPTE, participation in the survey was restricted to migrants who had lived in the destination city for over 6 months. Finally, 129,431 migrants were included in this analysis after excluding those with missing information on any study variables.

### Variables

CDPTE is the provision of essential health education services including prevention and control of CDs free of charge to migrants through the implementation of projects funded by the government to meet their basic needs [20]. CDPTE utilization among migrants was measured by the survey question “Have you received communicable disease prevention and treatment education in the past year?”. Binary: 1 for received CD prevention and treatment education, 0 for did not receive or no answer. The acceptance rate of CDPTE was calculated by dividing the number of participants who have received CDPTE (answering ‘yes’) by the total number of participants.

Explanatory variables were based on four categories, i.e., (1) predisposing factors that included demography (sex, and age), as well as social structure (marital status, and education attainment); (2) enabling factors, including both individual/family information (household monthly income, and average weekly working time), as well as the region information (survey region); (3) need factors, which entailed evaluated need (had health record, and contracted family doctor) as well as the perceived need (self-evaluation general health status); and (4) migration characteristics (length of migration and range of migration). The Survey region referred to the place to which participants migrated and was categorized based on the National Bureau of Statistics of the PRC for classifying regional economic growth [38].

### Statistical analysis

Descriptive statistical analysis was used to report demographic variables and methods of CDPTE, including frequencies, percentages, means, and standard deviations (SDs). Chi-square tests were performed to screen potential factors influencing CDPTE utilization across all variables [39]. For analysis of “acceptance methods of CDPTE ”, we screened 51,840 CDPTE migrants out of the 129,431 who had received health education. Thereafter, we statistically analyzed “acceptance methods of health education” to clarify the acceptance rates across different education methods. Thereafter, multivariate logistic regression analysis was performed, incorporating all influencing factors screened from the first step. This was to estimate the influencing factors of CDPTE utilization [40]. A list of explanatory variables is outlined in Table 1. All statistical analyses were performed by IBM Statistical Package for Social Science version (IBM-SPSS) 26.0, at a statistical significance level of *p-*value less than 0.05.

**Table 1:** The list of explanatory variables for analysis

Variables			levels
Predisposing	Demography	Sex	Female (Reference group); Male
		Age (years)	15–19 (Reference group); 20–29; 30–39; 40–49; ≥50
	Social structure	Marital status	Unmarried (Including divorced/widowed, reference group); Married (Including first marriage, remarriage, cohabitation)
		Education attainment	Primary school or below (Reference group); Middle school; High school or secondary; University or college and above
Enabling	Individual/family resources	Household monthly income (CNY)	< 4,000 (Reference group); 4,000–5,499; 5,500-7,499; 7,500-9,999; ≥10,000
		Average weekly working time (hours)	≤ 40 (Reference group); 41–69; ≥70
	Region resources	Survey region	East (Reference group); Central; West; Northeast
Need	Had health record		No (Reference group); Yes
	Contracted family doctor		No (Reference group); Yes
	Self-evaluation general health status		Good (Reference group); Other (Including fair; unwell)
Migration characteristics	Length of migration (years)		≤ 3 (Reference group); 4–6; 7–9; ≥10
	Range of migration		Inter-provincial (Reference group); Intra-provincial; Inter-country within the city

## Result

Table 2 shows the characteristics information of the sampled population. The sampled population included 129,431 migrants. Generally, male participants (51.4%) were more than females. The mean age of the participants was 37.78 (SD = 11.1), with most of them in the age range of 30 to 39 (34.4%). Their average level of education was low, with 80.9% of participants having no university or higher level of education. In addition, 83.7% of the participants were married, while 86.1% rated themselves as being healthy. Between 40 and 80% of the participants had an average monthly household income of 5,500 − 10,000 CNY (or US$863-1, 568). Above half of the participants (63.2%) worked more than 40 h a week. With regards to the destination, the majority of participants (44.3%) were in the eastern region, with the least number (7.2%) in the northeast region. As for migration characteristics, most participants were inter-provincial migrants (49.8%), with the duration of migration ≤ 3 years (38.5%). We also found that 87.6% of participants did not contact a family doctor, whereas 71.5% did not establish a health record.

Acceptance Of CDPTE Among Migrants with Different Characteristics.

In the full sample, only 40.1% of the participants received CDPE in their local communities. Among four major regions, the rate of CDPTE acceptance among migrants in the East, Central, West, and Northeast regions were 32.3%, 48.0%, 48.7%, and 31.2% respectively. Results of the Chi-square test revealed that CDPTE acceptance among migrants differed by sex, age, marital status, education attainment, household monthly income, average weekly working time, survey region, whether or not to have a health record, whether or not to contract a family doctor, self-evaluation general health status, length of migration, and range of migration, and the differences were statistically significant (*p* < 0.001) (Table 2).

**Table 2:** The utilization of CDPTE among the migrants by each explanatory variable (N = 129,431)

Variances	Total *N* (%)	Received CDPTE		Χ^2^	*p*
		Yes	No		
*Predisposing variables*					
Sex				81.138	< 0.001
Female	62,907 (48.6)	24,402 (38.8)	38,505 (61.2)		
Male	66,524 (51.4)	27,438 (41.2)	39,086 (58.8)		
Age (years)				197.365	< 0.001
15–19	2,391 (1.8)	1,003 (41.9)	1,388 (58.1)		
20–29	34,221 (26.4)	13,946 (40.8)	20,275 (59.2)		
30–39	44,550 (34.4)	18,366 (41.2)	26,184 (58.8)		
40–49	30,817 (23.8)	12,357 (40.1)	18,460 (59.9)		
≥ 50	17,452 (13.5)	6,168 (35.3)	11,284 (64.7)		
Marital status				35.041	< 0.001
Unmarried	21,089 (16.3)	8,832 (41.9)	12,257 (58.1)		
Married	108,342 (83.7)	43,008 (39.7)	65,334 (60.3)		
Education attainment				365.096	< 0.001
Primary school or below	21,367 (16.5)	7,435 (34.8)	13,932 (65.2)		
Middle school	54,606 (42.2)	21,934 (40.2)	32,672 (59.8)		
High school or secondary	28,724 (22.2)	12,391 (43.1)	16,333 (56.9)		
University or college and above	24,734 (19.1)	10,080 (40.8)	14,654 (59.2)		
***Enabling factors***					
Household monthly income (CNY)				246.985	< 0.001
< 4,000	20,145 (15.6)	8,277 (41.1)	11,868 (58.9)		
4,000–5,499	29,956 (23.1)	12,476 (41.6)	17,480 (58.4)		
5,500-7,499	27,036 (20.9)	11,166 (41.3)	15,870 (58.7)		
7,500-9,999	19,867 (15.3)	8,126 (40.9)	11,741 (59.1)		
≥ 10,000	32,427 (25.1)	11,795 (36.4)	20,632 (63.6)		
Average weekly working time (hours)				251.244	< 0.001
≤ 40	47,640 (36.8)	17,743 (37.2)	29,897 (62.8)		
41–69	45,643 (35.3)	19,160 (42.0)	26,483 (58.0)		
≥ 70	36,148 (27.9)	14,937 (41.3)	21,211 (58.7)		
Survey region				3574.560	< 0.001
East	57,307 (44.3)	18,516 (32.3)	38,791 (67.7)		
Central	23,818 (18.4)	11,425 (48.0)	12,393 (52.0)		
West	39,000 (30.1)	18,997 (48.7)	20,003 (51.3)		
Northeast	9,306 (7.2)	2,902 (31.2)	6,404 (68.8)		
***Need factors***					
Had health record				4925.862	< 0.001
No	92,522 (71.5)	31,471 (34.0)	61,051 (66.0)		
Yes	36,909 (28.5)	20,369 (55.2)	16,540 (44.8)		
Contracted family doctor				3231.547	< 0.001
No	113,357 (87.6)	42,097 (37.1)	71,260 (62.9)		
Yes	16,074 (12.4)	9,743 (60.6)	6,331 (39.4)		
Self-evaluation general health status				407.945	< 0.001
Good	111,432 (86.1)	45,863 (41.2)	65,569 (58.8)		
Other	17,999 (13.9)	5,977 (33.2)	12,022 (66.8)		
***Migration characteristics factors***					
Length of migration (years)				101.261	< 0.001
≤3	49,806 (38.5)	20,484 (41.1)	29,322 (58.9)		
4–6	28,523 (22.0)	11,521 (40.4)	17,002 (59.6)		
7–9	19,393 (15.0)	7,881 (40.6)	11,512 (59.4)		
≥ 10	31,709 (24.5)	11,954 (37.7)	19,755 (62.3)		
Range of migration				1257.647	< 0.001
Inter-provincial	64,483 (49.8)	22,720 (35.2)	41,763 (64.8)		
Intra-provincial	42,951 (33.2)	19,027 (44.3)	23,924 (55.7)		
Inter-country within the city	21,997 (17.0)	10,093 (45.9)	11,904 (54.1)		

Additionally, the accepted methods of health education were more diversified among the 51,840 migrants who received CDPTE, based on the proportion of education acceptance methods in descending order: 43,305 migrants (83.5%) received promotional materials; 38,052 migrants (73.4%) received health education bulletin boards/electronic displays; 28,606 migrants (55.2%) received health knowledge lectures; 25,741 migrants (49.7%) received community SMS/WeChat/Internet and other new media means; 18,648 migrants (36.0%) received public health consultation activities, and 9,823 migrants (18.9%) received individualized face-to-face consultation (Table 3).

**Table 3:** Approaches of CDPTE delivered to migrants

	Frequency	Percentage (%)
Promotional materials	43,305	83.5
Health education bulletin boards	38,052	73.4
Health knowledge lectures	28,606	55.2
Community SMS/WeChat/Internet and other new media means	25,741	49.7
Public health consultation activities	18,648	36.0
Individualized face-to-face consultation	9,823	18.9

### Multivariate logistic regression

Multivariate logistic regression results indicated that male migrants (OR = 1.100, 95% CI:1.074, 1.127) and unmarried status (OR = 1.154, 95% CI:1.113, 1.197) were more likely to have received CDPTE. Similarly, migrants aged between 30–39, and 40–49 years in the past year had 1.125- (OR = 1.125, 95% CI:1.080, 1.173) and 1.148-fold (OR = 1.148, 95% CI:1.101, 1.196) higher chances of receiving CDPTE, compared to those who were above 50 years. Migrants with University or college education and above, high school or secondary, and middle school were 1.278- (OR = 1.278, 95% CI:1.221, 1.337), 1.352- (OR = 1.352, 95% CI:1.297, 1.409), and 1.227-times (OR = 1.227, 95%CI:1.183, 1.272) more likely to receive CDPTE in the past year, relative to those with primary school education or below. In addition, migrants with an average monthly household income of CNY7,500-9,999 (or US$1,176-1,567) in the past year had a 1.061-fold (OR = 1.061, 95% CI:1.015, 1.109) higher chance of receiving CDPTE, compared to those with an average monthly household income of less than 4,000 CNY (or US$627). On the other hand, those with an average monthly household income of more than 10,000 CNY (or US$1, 568) had a 0.945-fold (OR = 0.945, 95% CI:0.906, 0.985) less chance of receiving CDPTE. Migrants with an average weekly working time of more than 70, and 41–69 h were 1.174 times (OR = 1.174, 95% CI:1.138, 1.211) and 1.181 times (OR = 1.181, 95% CI:1.148, 1.214) more likely to receive CDPTE, compared with those who had an average weekly working time less than 40 h. Migrants flowing to the central and western regions were 1.507 times (OR = 1.507, 95% CI:1.456, 1.560) and 1.826 times (OR = 1.826, 95% CI:1.774, 1.880) more likely to receive CDPTE than their counterparts from the eastern region. On the other hand, those flowing to the northeastern region had a 0.897-fold (OR = 0.897, 95%CI:0.853, 0.943) lower chance of receiving CDPTE. Additionally, migrants with health records (OR = 1.904, 95% CI:1.849, 1.960) as well as those who contracted a family doctor (OR = 1.537, 95% CI:1.477, 1.601) were more likely to receive CDPTE. The chances of receiving CDPTE decreased by 25.0% in poor health status (OR = 0.750, 95% CI: 0.723, 0.778), compared to participants with self-evaluated good health status. Migrants with 4–6, and over 10 years of residence were 0.957 times (OR = 0.957, 95% CI:0.928, 0.987) and 0.947 times (OR = 0.947, 95% CI:0.918, 0.978) less likely to receive CDPTE, compared to those with 3 or fewer years of residence. Moreover, migrants with intra-provincial and inter-country movement within the city were 1.180 times (OR = 1.180, 95% CI:1.148, 1.212) and 1.187 times (OR = 1.187, 95% CI:1.146, 1.229) more likely to receive CDPTE compared to their Inter-provincial counterparts (Table 4).

**Table 4:** Multivariate logistic regression analysis of the explanatory variables of CDPTE utilization

Variables in the equation	*B* (SE)	Wald	OR (95%CI)	*P*
*Predisposing variables*				
Sex (Ref = Female)				
Male	0.096 (0.012)	61.204	1.100 (1.074, 1.127)	< 0.001
Age (Ref = ≥ 50)				
15–19	0.046 (0.049)	0.878	1.047 (0.951, 1.154)	0.349
20–29	0.038 (0.023)	2.745	1.039 (0.993, 1.087)	0.098
30–39	0.118 (0.021)	31.802	1.125 (1.080, 1.173)	< 0.001
40–49	0.138 (0.021)	42.760	1.148 (1.101, 1.196)	< 0.001
Marital status (Ref = Married)				
Unmarried	0.143 (0.019)	58.763	1.154 (1.113, 1.197)	< 0.001
Education attainment (Ref = Primary school or below)				
Middle school	0.204 (0.018)	123.735	1.227 (1.183, 1.272)	< 0.001
High school or secondary	0.302 (0.021)	206.576	1.352 (1.297, 1.409)	< 0.001
University or college and above	0.245 (0.023)	112.664	1.278 (1.221, 1.337)	< 0.001
***Enabling factors***				
Household monthly income (Ref = <;4,000)				
4,000–5,499	0.016 (0.020)	0.654	1.016 (0.978, 1.056)	0.419
5,500-7,499	0.033 (0.021)	2.606	1.034 (0.993, 1.077)	0.106
7,500-9,999	0.059 (0.023)	6.825	1.061 (1.015, 1.109)	0.009
≥ 10,000	-0.057 (0.021)	7.062	0.945 (0.906, 0.985)	0.008
Average weekly working time (Ref = ≤ 40)				
41–69	0.166 (0.014)	132.406	1.181 (1.148, 1.214)	< 0.001
≥ 70	0.161 (0.016)	103.180	1.174 (1.138, 1.211)	< 0.001
Survey region (Ref = East)				
Central	0.410 (0.018)	535.782	1.507 (1.456, 1.560)	< 0.001
West	0.602 (0.015)	1647.828	1.826 (1.774, 1.880)	< 0.001
Northeast	-0.108 (0.026)	17.962	0.897 (0.853, 0.943)	< 0.001
***Need factors***				
Had health record (Ref = No)				
Yes	0.644 (0.015)	1855.456	1.904 (1.849, 1.960)	< 0.001
Contracted family doctor (Ref = No)				
Yes	0.430 (0.021)	437.267	1.537 (1.477, 1.601)	< 0.001
Self-evaluation general health status (Ref = Good)				
Other	-0.288 (0.018)	242.946	0.750 (0.723, 0.778)	< 0.001
***Migration characteristics factors***				
Length of migration (Ref = ≤ 3)				
4–6	-0.044 (0.016)	7.794	0.957 (0.928, 0.987)	0.005
7–9	-0.014 (0.018)	0.568	0.986 (0.952, 1.022)	0.451
≥ 10	-0.054 (0.016)	11.128	0.947 (0.918, 0.978)	< 0.001
Range of migration (Ref = Inter-provincial)				
Intra-provincial	0.165 (0.014)	139.828	1.180 (1.148, 1.212)	< 0.001
Inter-country within the city	0.171 (0.018)	93.503	1.187 (1.146, 1.229)	< 0.001

Footnote: Full regression models significantly explained the variance in CDPTE utilization among the migrants (*p* = 0.000)

The model explained between 7.0% (Cox and Snell R square) and 9.5% (Nagelkerke R square) of the variance in the utilization of CDPTE

## Discussion

In this study, we evaluated utilization of CDPTE among migrants, then applied the Anderson health service utilization model to assess the major factors affecting its utilization, to better facilitate their health education service utilization. Our results revealed that 40.1% of the recruited migrants received CDPTE in their local communities. This was higher than the average level of health literacy for CD (17.05%) reported in the same year in China’s Resident Health Literacy Monitoring Report [41]. This finding corroborated that of previous studies. For instance, a study in Shanghai revealed that the rate of health education utilization on CDs among migrants was only 30% in 2014 [42], whereas a 2017 study found that only 33.7% of migrants received education on tuberculosis prevention [43]. According to the 13th Five-Year Plan for the Management of Health and Family Planning Services for the National Migrant Population, the comprehensive health plan targets to achieve over 95% coverage of health education for migrants by 2020 [44]. In the present study, the overall acceptance rate of CDPTE among migrants largely fell behind this target. Moreover, CDPTE acceptance among migrants was still dominated by traditional communication media, including promotional materials (paper, film, and television), as well as bulletin boards/electronic displays, followed by health knowledge lectures. Notably, the use of more convenient approaches for elderly migrants, including face-to-face consultations and health lecture consultations, was scarce. Although the Internet and other new media platforms are currently the main approaches for information dissemination, our results revealed that only 49.7% of migrants received CDPTE using new media including WeChat. This indicates that the use of cell phones and other new media platforms to educate migrants on occupational disease prevention and treatment is not fully popularized. In recent years, the Chinese government has launched a series of policies and measures for the prevention and treatment of CDs, which have consequently played an important role in controlling the CD epidemic in the country. However, China still faces severe pressure and remains a long way from delivering health education services, particularly those for effective prevention and treatment of CDs. Therefore, there is an urgent need to strengthen relevant health education services to improve the availability of CD health literacy using multiple educational contents and multidimensional educational approaches. Policymakers should continually develop targeted interventions for improved utilization of basic public health services among migrants.

Our results revealed that age and literacy are the major factors influencing CDPTE, consistent with previous studies [45]. Among them, migrants aged between 30 and 49 years were more likely to receive CDPTE, whereas those aged 50 years and above were less likely to receive CDPTE. This may be attributed to the fact that older migrants have declining memory and worse health status, hence less motivation to receive knowledge about CDs. Moreover, migrants with junior high school education and above were more likely to receive CDPTE, indicating the limited effect of the current organized and conducted communicable disease health education activities among older and lowly-educated groups. Similarly, a study conducted in China showed that a lower level of education was associated with poorer CD knowledge among migrants [46]. Firstly, migrants with higher education status had stronger health awareness and more active demand for public health services. Besides, they could acquire reliable health information from various sources and had better access, understanding, and mastery of education on CD prevention and treatment. In contrast, lowly-educated migrants were socioeconomically disadvantaged, thus having poor access to health education [18], inadequate social support network, and living in areas with higher exposure to risks [47]. In addition, they have limited access to public health resources, predisposing them to CDs. Our results further demonstrated that CDPTE utilization is higher among male than female migrants. Notably, female migrants received limited health education services, primarily on reproductive health as well as maternal and child health [48], and were less aware than their male counterparts on CDPTE. This was consistent with results from previous studies. We attribute the discrepancies to potential selection bias due to the relatively young migrants. Moreover, married migrants were less likely to receive CDPTE compared to their unmarried counterparts. This perhaps because most of the migrants investigated were married and took the responsibility for taking care of their child(ren) and parent(s), hence their relatively high demand for health education on child and adolescent health as well as pregnancy care, prevention, and treatment of non-communicable disease (NCD) [49]. Furthermore, unmarried migrants have greater life and mental stress and are more sensitive to health risks, including their communicable diseases, than those with more stable marital status, thus are more likely to choose to receive CDPTE. Collectively, these results show that CDPTE should focus on females, the older generation, and lower-educated groups. The results also affirm the need for precise health education work and differentiated policy development, which should consider the different characteristics and specific needs of the migrants. At present, posters, videos, and lectures are the most widely used forms of health education. However, subjects do not explicitly understand text-based promotional materials, because some migrants have declining audiovisual function/low literacy rates. Given the characteristics of this special group of people, targeted, interesting and rich forms of CDPTE and promotion activities, including health education lectures and case-based health education, should be used as much as possible. Educators are advised to use the local language in providing concise and popular teaching materials on communicable disease knowledge and skills. Moreover, there is a need to sufficiently explain the advantages of new media to strengthen online education, promote popularization, and application of WeChat and other health websites. These can not only improve the coverage of CDPTE but also continuously improve the effectiveness of education in a targeted manner, thereby helping a majority of the migrants.

We further revealed that household monthly income, the average weekly working hours, and the survey region of enabling resources are significant predictors of CDPTE uptake. This was in contrast to findings from previous studies in China [31]. Generally, migrants with low average monthly household income and those working less than 40 h a week are predominantly engaged in occupations such as production, construction, and transport with high labor intensity, poor pay, and stability [50, 51]. Studies have shown that migrant workers compensate the majority of the migrants [52]. The vast majority of these individuals are overwhelmingly employed in the 3D (dirty, dangerous, and degrading) sector [31], with intense and stressful work, relatively poor working conditions, and low social status, predisposing them to a greater risk of contracting CDs during work, thus may have deficiencies in hygiene practices and behaviors exacerbating infection by common CDs. This group lacks group health education from their workplace, has limited access to health education, and has low group literacy in CD control [53]. Additionally, migrants with low average monthly household incomes have life stresses or other difficulties, focusing more on improving their living conditions and that of their families as well as paying less attention to health services. Moreover, these migrants are more likely to congregate in places with relatively poor living conditions to ease the cost of living [54]. Our findings are also supported by the previous study that 1.27% of migrants still live in shacks, basements, and other types of housing [55]; this is where poor lighting and ventilation, as well as high levels of rodents, ants, and mosquitoes, create conditions conducive for the spread of bacteria and viruses causing the development of communicable symptoms among migrants. Furthermore, individuals working longer hours are mostly young migrants, with relatively higher incomes, and more stable jobs. Employers may be more concerned about the importance of health education, providing more diverse health resources and services to their employees in the workplace and work environment. A 2013 study in Shanxi Province revealed that regular training of migrants on key CDs, including HIV, hepatitis B, and tuberculosis, could improve their willingness to receive CDPTE thereby promoting healthy behaviors to a certain extent [56]. Interestingly, migrants with an average monthly household income of more than 10,000 CNY (US$ 1,568) were negatively associated with the utilization of CDPTE. On the other hand, those with higher incomes appeared to overlook their health status, and the heavy workloads and situational stress reduce their need to access health information, specifically for communicable diseases, a phenomenon that provided them less time to utilize public health services [42]. At the same time, our study findings suggest that migrants in the East and North East regions were less likely to receive CDPTE, which should be attributed to the barriers developed by the household registration system. Eastern regions with a higher density of migrants, which are economically developed with abundant healthcare and financial resources, have a high threshold for household registration to restrict the movement of several migrants to the local area and prevent an increased burden on public health services [57, 58]. A previous study revealed that migrants hardly obtained permanent urban household registration in the destination city, particularly in more economically developed East regions [32, 59], where their health did not benefit from local public health service provisions. On the other hand, the central government mainly provides subsidies for basic public health services to the Central and Western regions, however, only 10–50% of the total subsidies are allocated to the Eastern and Northeastern regions [60]. Thirdly, the pace of economic development in the Northeast is relatively slow, health resources are basically at a relatively low level, and health education resources are supplied at a low level of efficiency and equity [61], hence difficult to meet the demand for public health services for the migrants. This discrepancy has caused poor accessibility of resources for their health education services. According to the above findings, health education resources should be appropriately promoted to migrants, with their workplaces and residences being key promotional units for the dissemination of knowledge and preventive behaviors about common and vulnerable CDs including influenza, AIDS, and tuberculosis. Relevant government departments should break the regional restrictions on basic public health services, and promote equality and accessibility of regional health education resources, including communicable disease control and public health services through policy protection and financial support to realize cross-regional resource and information sharing.

Migration characteristics substantially promote inequality in health education utilization among migrants. Our results revealed that intra-provincial migrants were more likely to receive CDPTE compared to their inter-provincial counterparts; this indicates that the range of migration was linked to the utilization of health education services for migrants. This finding is consistent with the results by Zhang et al., who discovered that inter-province migration significantly reduced the likelihood of using health education compared to that within a province [32]. This is perhaps because inter-provincial migration covers a greater distance than that intra-provincial migration and inter-country within the city, causing major differences in social culture, daily language use, and living habits from their original place of residence [62, 63]. These circumstances increase the difficulty of cultural recognition and social integration of migrants, directly reducing their subjective sense of belonging. In addition, inter-provincial re-employment may face difficulties regarding low starting points for job remuneration and poor stability [32], as well as implying a separation of migrants from their family location, a weak material base, and a lack of social security in the city of inflow. Thus, migrants are less stable and less dependent on resources for health education services including CD prevention and treatment. In addition, the length of migration inhibits CDPTE utilization. Specifically, in the early stages of migration, the departure from the familiar social network, the low level of social adaptation, and the unfamiliar living and working environment have a greater negative impact on migrants, both physically and psychologically. This causes a greater demand for community-based basic health care and public health services [64, 65]. Migrants who had lived in the inflow area for a longer period were marginalized in several ways. They also revealed a lower level of social integration and comparatively poor social and economic status and did not yet have full access to a range of public health services, employment, pension, health education, and medical insurance provided by the location [19, 59]. One previous study revealed that migrants experienced marginalization, including poor working and living environments, as well as weak health literacy, and inadequate access to social security and health benefits [32]. The longer the migrants live, the more familiar they become with the environment and the people in the inflow area, resulting in a distrustful attitude towards the level of medical technology and public health services in the grassroots community health service centers and low utilization of public health services. Moreover, related reports have indicated that migrants have misconceptions about symptoms related to communicable diseases, transmission routes, prevention and intervention measures, and consequences of infection, whereas incidences and transmission rates of communicable diseases appeared high [20]. In recent years, China has developed a number of policy documents on health management for migrants, and clear arrangements have been established to improve their basic public health services [11, 66]. However, a few policies and measures are essentially more “management” than “service”, with a weak sense of service and a strong sense of compulsion, with little significance for improving the health status of migrants [67]. At the same time, the relevant policies are not well targeted, and places lack specific implementation plans and supervision, and assessment. Therefore, targeted interventions are necessary to improve the motivation and initiative of migrants to receive CDPTE. Dissemination of educational content on diseases including tuberculosis, and pneumonia among other communicable diseases, coupled with CD prevention and treatment strategies, should be performed in a more convenient and friendly way.

Several other issues are worth discussing. First, our finding indicates that migrants who rate themselves as healthy were more likely to receive CDPTE, due to their higher health awareness, higher perception and acceptance of health information, promoting their health-promoting behaviors and decisions, and were more proactive in receiving CDPTE [68]. Secondly, with better use of health services including health records and family doctors, migrants were more likely to accept CDPTE. This is because migrants who assess themselves as healthy have higher health awareness, higher perception, and acceptance of health information, which facilitates their health-promoting behavior and decision-making, and greater initiative in accepting CDPTE. Migrants who have established health records and signed up with a family doctor are more likely to choose to settle for the long term. For the sake of long-term living and development in the place of inflow [32, 69], migrants will actively seek services including health education to maintain and promote their health and safety, and have better cooperation with policies related to basic public health services. Furthermore, by establishing health records and contracting family doctors, migrants can increase their attention to health management and health behavioral modification, hence a greater probability of participating in health promotion activities for the prevention and control of CDs. These services have been identified as important tools for the public health education of migrants and promote the equalization of basic public health services among the population [49]. The designated policy predicted that the contracted service for family doctors will achieve full coverage and the rate of health records may exceed 80% by 2020 [66, 70]. However, in this study, the contraction rate of a family doctor was 12.4%, and the rate of health records was 28.5%, which were below the national recommended rates. Therefore, increasing the use of health records and family doctor services can indirectly improve the health status of migrants by increasing their knowledge of infectious diseases and maintaining healthy habits, which is important for health promotion and beneficial for improving the quality of health education.

## Limitations of the study

The present study has several limitations. First, a cross-sectional design was adopted, which does not allow the determination of causality, trends, or long-term effects of CDPTE among migrants. Secondly, secondary data from publicly available datasets were analyzed, limiting the number of variables we could use due to existing data limitations. Moreover, we excluded other factors that may affect the utilization of CDPTE, including the supply of communicable disease control education services. Additional variables should be identified in future studies to explain the dynamic and cyclical causality of the Anderson health service utilization model. Thirdly, we did not consider the prevalence of CDs and even the types of CDs. Lastly, utilization of CDPTE was measured as a dichotomous variable (i.e., groups that received or did not receive CDPTE), instead of measuring the intensity of CDPTE applied. The mediating and moderating effects between independent variables were also not investigated. Despite these limitations, this work presents reference data for future longitudinal analyses.

## Conclusion

In conclusion, this study shows that acceptance of CDPTE among migrants is poor and health education should be provided to this group. Besides the traditional media, it is important to provide education on communicable disease prevention and treatment through current media platforms including the Internet and WeChat, to increase awareness and stimulate the active participation of migrants by advocating behavior change. Several predisposing factors (sex, age, marital status, education attainment), enabling resources (household monthly income, average weekly working time, survey region), need factors (having health record, contracting family doctor, self-evaluation general health status), and migration characteristics factors (length of migration and range of migration) were identified as the primary factors influencing utilization of CDPTE among migrants in China. The health of migrants is not just a personal issue, but a concern of the whole society where they live. Improving the level of CDPTE among migrants requires the collective participation of many sectors. Therefore, policies targeting the implementation of CDPTE among migrants should be improved for specific regions and vulnerable groups to improve the use of CDPTE among migrants. This study also underscores the need for more diverse CDPTE programs for migrants. Moreover, the government should increase financial support to improve the provision of health education services including continuous improvement of health records and family doctor service specifications.

## Data Availability

The data used in this study are from the Migrant Population Service Center, National Health Commission of China (NHCC). The datasets used and/or analyzed during the current study are available from the corresponding author on reasonable request.

## Abbreviations

CMDS: China Migrants Dynamic Survey
COVID-19: Coronavirus disease 2019
CD: Communicable disease
NCD: Chronic non-communicable disease
HIV: The human immunodeficiency virus
SD: Standard Deviation
OR: Odds ratio
CI: Confidence interval
PEN: Predisposing, enabling, and need variances
CNY: Chinese Yuan
